# Vitamin D receptor gene polymorphisms and its interactions with environmental factors on renal cell carcinoma risk

**DOI:** 10.1186/s41021-021-00185-3

**Published:** 2021-05-18

**Authors:** Tian Jianhai, Lv Jian, Zhang Long, Wang Wei, Zhang Shumao, Wang Yiming, Li Xiaojuan

**Affiliations:** Department of Urology, Linyi cancer hospital, No.6 Lingyuan East Street, Lanshan District, Shandong Province Linyi CIty, China

**Keywords:** Alcohol drinking, Renal cell carcinoma, Vitamin D receptor, Single nucleotide polymorphisms, Interaction, Smoking

## Abstract

**Aims:**

We designed a case-control study to investigate the effect of vitamin D receptor gene (VDR) gene single nucleotide polymorphisms (SNPs) and possible gene- environment interaction on the susceptibility of renal cell carcinoma (RCC).

**Methods:**

Generalized multifactor dimensionality reduction (GMDR) was used to find out the interaction combinations between SNPs and environmental factors, including gene- gene synergy and gene environment synergy effect. Logistic regression was used to analyze the correlation between the four SNPs in VDR gene and RCC, and the significant interaction combinations found by GMDR model were analyzed by hierarchical analysis.

**Results:**

The genotype distribution of the control group was in accordance with Hardy- Weinberg equilibrium. Logistic regression analysis showed that the risk of RCC in VDR-rs7975232 A allele carriers was significantly higher than that of CC genotype carriers (CA + AA vs. CC), adjusted OR (95 % CI) = 1.75 (1.26–2.28). We used GMDR model to screen the best synergistic model between the four SNPs of VDR gene and smoking and drinking. We found a significant two locus model (*P* = 0.0010) involving rs7975232 and smoking. The cross- validation consistency of the two- locus model was 10/ 10, and the accuracy was 60.72 %. Compared with non-smokers with rs7975232 -CA or AA genotype, smokers with rs7975232 -CC genotype had the highest risk of RCC, or (95 % CI) = 2.23 (1.42–3.09), after adjustment for covariates.

**Conclusions:**

We found that the A allele of rs7975232 within VDR gene, interaction between rs7975232 and smoking were all associated with increased RCC risk.

## Introduction

Renal cell carcinoma is one of the most common malignant tumors in urinary system and one of the most lethal tumors [1]. There were 40,3262 newly diagnosed tumors in kidney, and 17, 5098 persons died from this cancer worldwide in 2018 [2]. And in China, there are too many new kidney cancer cases died from kidney cancer in 2016 [3]. Both the incidence and the corresponding mortality rate have been steadily increasing over the past several years [4]. Several risk factors related RCC risk were reported previously, including smoking, obesity, hypertension, acetaminophen and viral hepatitis [5–7]. Approximately, 4 % of all RCC are hereditary as well [8].

Previous epidemiological and biological studies have shown that vitamin D levels have a significant impact on the occurrence and development of cancer [9]. Vitamin D receptor (VDR) genetic polymorphisms, which located on chromosome 12q12–q14, could control Vitamin D activity, have been reported to be associated with several diseases [10–12]. The name of each SNP in VDR is based on the restriction site originally used to identify it. Such as *FokI* and *BsmI*, as defined by the endonucleases *FokI* and *BsmI*, respectively, have been most frequently studied in the previous studies. Previously, several studies [13–16] have been performed to evaluate the association between VDR polymorphisms and RCC risk in different populations, including several meta- analysis studies; however, the results of these studies were conflict. In addition, previous studies have shown that the occurrence and development of RCC is a multi-step, complex and complex process involving multiple environmental factors, genetic factors and the synergy between genetic factors and environmental factors [17, 18]. However, up to now, there is no study on the relationship between VDR gene and environmental factors and the risk of renal cell carcinoma. Therefore, we designed a case-control study to investigate the effect of VDR gene SNPs and possible gene- environment interaction on the susceptibility of RCC.

## Materials and methods

### Subjects

A total of 1101 participants (634 males, 467 females) were selected, including 366 RCC patients and 735 control participants. All cases were primary renal cell carcinoma, and they were diagnosed by histopathology in our hospital. Those cases who had received any radiotherapy, chemotherapy or with any type of cancer were excluded from our study. Controls were cancer-free and were age- (less than 3 years) and sex-matched (nearly 1:2 matched) to patients. And those with any type of cancer, coronary heart disease, stroke and others history of serious diseases were excluded from control group. The subjects will participate in our questionnaire survey to obtain their general demographic information, lifestyle information and family history. Current drinking is defined as people who drink more than one type of alcoholic beverage per month, and the rest are non- drinkers. Current smokers were defined as those who smoked at least 100 cigarettes and still did not quit smoking at the time of filling out the questionnaire; people without a history of smoking were considered never to smoke. Each participant understood the process of the study and signed a written informed consent before the start of the study.

### Genotyping

A total of 4 SNPs within VDR gene were selected for genotyping, including: rs7975232, rs2228570, rs1544410 and rs731236. Genomic DNA from participants was extracted from EDTA-treated whole blood, using the DNA Blood Mini Kit (Qiagen, Hilden, Germany) according to the manufacturer’s instructions and stored at -20 °C until use. The polymerase chain reaction-restriction fragment length polymorphism (PCR-RFLP) was performed to detect the polymorphisms of these 4 SNPs (Table 1), according previous study [19].

**Table 1:** Description and primers for 4 SNPs within VDR gene used for PCR analysis

SNP	Chromosome	Functional Consequence	Annealing temp.	Alleles	Restriction enzymes	Primer sequences
*FokI* rs2228570	12:47,879,112	Missense	72.5 °C	C > T	*FokI*	F: GCACTGACTCTGGCTCTGAC R: ACCCTCCTGCTCCTGTGGCT
***ApaI*** **rs7975232**	12:47,845,054	Intron variant	66.0 °C	C > A	*ApaI*	F: CAGAGCATGGACAGGGAGCAA R: GCAACTCCTCATGGCTGAGGTCTC
*TaqI* rs731236	12:47,844,974	Coding sequence variant, synonymous variant	51.9 °C	T > C	*TaqI*	F:5’- CCTGTGCCTTCTTCTCTAT − 3’ R: 5’- CTAGCTTCTGGATCATCTTG- 3’
*BsmI* rs1544410	12:47,846,052	Intron variant	50.5 °C	G > A	*BsmFI*	F: 5’- ATATAGGCAGAACCATCTCT − 3’ R: 5’- TCTGAGGAACTAGATAAGCA − 3’

### Statistical analysis

The mean and standard deviation (SDs) of normal distribution continuous variables were calculated, and the differences of continuous variables between case group and control group were compared by Student t test. For categorical variables, we calculated the percentages and compared the differences between the case group and the control group using the chi square test. Generalized multifactor dimensionality reduction (GMDR) was used to find out the interaction combinations between SNPs and environmental factors, including gene- gene synergy and gene environment synergy effect, and in this model, some factors could be selected for adjustment, including gender, age, hypertension, diabetes, smoking or alcohol drinking and BMI. Logistic regression was used to analyze the correlation between the four SNPs in VDR gene and RCC, and the significant interaction combinations found by GMDR model were analyzed by hierarchical analysis. The P values listed in all the results were double tailed, and those with P values less than 0.05 were considered to be statistically significant.

## Results

A total of 1101 eligible subjects (634 males and 467 females) were selected, including 366 eligible RCC patients and 735 normal control subjects. The average age of all subjects was 63.2 ± 14.2 years. Table 2 lists the general clinical and demographic characteristics of different subjects in the case and control groups. There was no significant difference in age, body mass index and male ratio between the case group and the control group. However, the prevalence of smoking and drinking, hypertension and diabetes in the case group were significantly higher than those in the control group.

**Table 2:** General characteristics of 1101 study participants in RCC patients and controls

Variables	Case group (*n* = 366)	Normal group (*n* = 735)	*p-*values
Age (year) (Means ± SD)	62.8 ± 12.5	63.5 ± 13.2	0.399
BMI (kg/m^2^) (Means ± SD)	23.6 ± 8.6	23.2 ± 8.9	0.478
Gender			0.675
Males, N (%)	214 (58.5)	420 (57.1)	
Females, N (%)	152 (41.5)	315 (42.9)	
Smoking			0.0084
Current smokers, N (%)	100 (27.3)	149 (20.3)	
Never or past smokers, N (%)	266 (72.7)	586 (79.7)	
Alcohol consumption			0.029
Current drinkers, N (%)	117 (32.0)	189 (25.7)	
Never or past drinkers, N (%)	249 (68.0)	546 (74.3)	
Diabetes, N (%)	59 (16.1)	74 (10.1)	0.004
Hypertension, N (%)	105 (28.7)	168 (22.9)	0.035
Family history of cancer N (%)	60 (16.4)	-	

The genotype distribution of the control group was in accordance with Hardy Weinberg equilibrium. The A allele frequency of rs7975232 in RCC patients was 29.9 %, which was significantly higher than that in controls (20.0 %). Logistic regression analysis showed that the risk of RCC in vdr-rs7975232 A allele carriers was significantly higher than that of CC genotype carriers (CA + AA vs. CC), adjusted OR (95 % CI) = 1.75 (1.26–2.28). However, we also found that rs2228570, rs1544410 and rs731236 were not significantly associated with RCC risk, adjusted for covariates. (Table 3)

**Table 3:** The association between 4 SNPs within VDR gene and RCC risk

SNP	Genotypes and Alleles	Frequencies N (%)		OR (95 %CI) ^a^	HWE test for controls
		Controls (*n* = 735)	Cases (*n* = 366)		
rs2228570 (*FokI*)					0.997
	CC	432 (58.8)	191 (52.2)	1.00 (ref)	
	CT	263 (35.8)	143 (39.1)	1.32 (0.91–1.83)	
	TT	40 (5.4)	32 (8.7)	1.55 (0.72–2.42)	
	CT + TT	303 (41.2)	175 (47.8)	1.39 (0.86–1.93)	
	Allele, T (%)	343 (23.3)	207 (28.3)		
rs7975232 (*ApaI*)					0.289
	CC	475 (64.6)	182 (49.7)	1.00 (ref)	
	CA	226 (30.7)	149 (40.7)	1.63 (1.21–2.04)	
	AA	34 (4.6)	35 (9.6)	2.02 (1.47–2.62)	
	CA + AA	260 (35.4)	184 (50.3)	1.75 (1.26–2.28)	
	Allele, A (%)	294 (20.0)	219 (29.9)		
rs731236 (*TaqI*)					0.735
	TT	460 (62.6)	200 (54.6)	1.00 (ref)	
	TC	241 (32.8)	139 (38.0)	1.24 (0.92–1.65)	
	CC	34 (4.6)	27 (7.4)	1.31 (0.81–1.86)	
	TC + CC	275 (37.4)	166 (45.4)	1.26 (0.89–1.69)	
	Allele, C (%)	309 (21.0)	193 (26.4)		
rs1544410 (*BsmI*)					0.655
	GG	466 (63.4)	211 (57.7)	1.00 (ref)	
	GA	236 (32.1)	130 (35.5)	1.31 (0.87–1.79)	
	AA	33 (4.5)	25 (7.8)	1.45 (0.78–2.15)	
	GA + AA	269 (36.6)	155 (42.3)	1.34 (0.84–1.87)	
	Allele, A (%)	302 (20.5)	180 (24.6)		

^a^Adjusted for gender, age, hypertension, diabetes, smoking, alcohol drinking and BMI

**Fig. 1:**
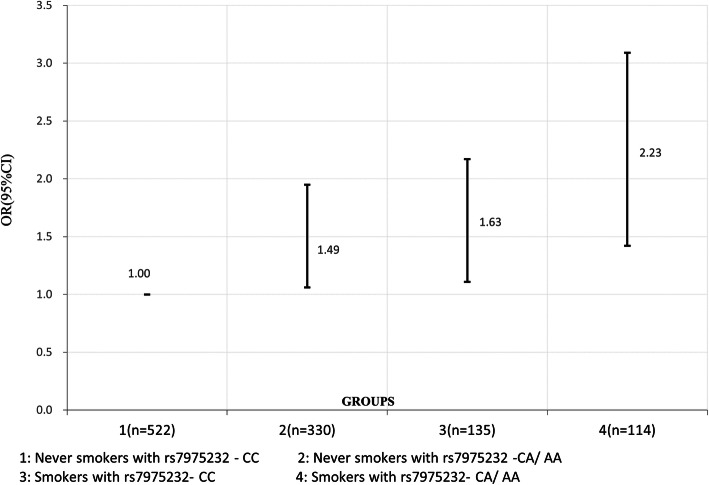
Hierarchical analysis for rs7975232 - alcohol drinking interaction using logistic regression. Hierarchical analysis for rs7975232- current smoking interaction using logistic regression

We used GMDR model to screen the best synergistic model between the four SNPs of VDR gene and smoking and drinking. Table 4 lists the best interaction combinations in each dimension of GMDR analysis. We found a significant two locus model (*P* = 0.0010) involving rs7975232 and smoking. The cross- validation consistency of the two- locus model was 10/ 10, and the accuracy was 60.72 %. However, we did not find any significant synergistic effect combination in GMDR model of SNP and alcohol drinking interaction. We also used logistic regression to analyze the synergistic effect between VDR-rs7975232 and smoking. We found that compared with non-smokers with rs7975232 -CA or AA genotype, smokers with rs7975232 -CC genotype had the highest risk of RCC, or (95 % CI) = 2.23 (1.42–3.09), after adjustment for covariates (Fig. 1).

**Table 4:** GMDR analysis on the best gene–environment interaction models

Locus no.	Best combination	Cross-validation consistency	Testing accuracy	*p-values*
Gene- alcohol drinking interactions^a^				
2	1, 5	9/10	0.572	0.055
3	1, 2, 5	7/10	0.540	0.172
4	1, 2, 4, 5	6/10	0.423	0.377
5	1, 2, 4, 3, 5	6/10	0.496	0.426
Gene- smoking interactions^b^				
2	1, 6	10/10	0.607	0.001
3	1, 3, 6	8/10	0.540	0.172
4	1, 3, 2, 6	7/10	0.487	0.623
5	1, 3, 2, 4, 6	6/10	0.478	0.989

^a^ Adjusted for gender, age, hypertension, diabetes, smoking and BMI

^b^Adjusted for gender, age, hypertension, diabetes, drinking and BMI

rs7975232, rs2228570, rs1544410, rs731236, current alcohol drinking and current smoking were symbolized as 1–6, respectively

## Discussion

In our study, we found that the A allele of **rs7975232** within VDR gene were significantly associated with increased RCC risk. However, we also found that rs2228570, rs1544410 and rs731236 were not significantly associated with RCC risk, adjusted for covariates. Several previous studies have reported the association between polymorphisms in VDR SNPs and risk of cancer, such as breast cancer and its survivors [20], colorectal cancer [21], lung cancer [22], advanced gallbladder cancer [23], bladder cancer [24], gastric cancer [25] and oral cancer [26]. To date, limited number of studies on relationship between *VDR* SNPs and RCC risk were performed, in addition, these previous studies could not obtain consistent results. Some previous studies have verified the relationship between genetic variation of *VDR* and RCC risk. Obara et al. [27] performed a case- control study suggested that AA genotype of ApaI locus in VDR gene may be a risk factor and poor prognosis factor of renal cell carcinoma in Japanese population. Arjumand et al. [13] indicated that *FokI* FF and *VDR* BB increased the cumulative risk of RCC by 1.87 times. Ou et al. [14] performed a case- control study suggested that the minor alleles of *ApaI, Fok1, BsmI* BB genotype, Fok1 gene were statistically associated with the susceptibility to RCC in Asians. Lin et al. [16] suggested that ApaI gene polymorphism and *Fok1* FF genotype were associated with RCC susceptibility in Asians. Yang et al. [19] also indicated that VDR ApaI gene mutation was associated with increased risk and susceptibility to RCC in Chinese Han population. Recently, a meta- analysis [15] obtained similar results with fore- mentioned studies. In current study, we did not find any relationship of *FokI, TaqI* and BsmI with RCC risk, but we found a significant relation between ApaI and RCC risk. Our results are inconsistent with other studies mentioned earlier, which may be due to the differences in genetic background, PD definition, sample size and statistical ability of the investigated population. In addition, these inconsistent results are also affected by multiple genetic heterogeneity, population mixing, gene environment and gene- gene interactions.

RCC susceptibility was influenced not only by environmental factors, genetic factors, but also by the gene- environment interactions. Previous studies have reported several risk factors significantly associated with RCC susceptibility, including hypertension, active or passive smoking, alcohol consumption and obesity [17, 28, 29]. Among these factors, smoking and drinking are the two major modifiable risk factors for RCC. In this study, the smoking and drinking rates of RCC patients were significantly higher than those of the control group. Therefore, six variables, including four SNPs and two environmental factors (current smoking and current drinking) were included in the GMDR model, we found a significant two locus model (*P* = 0.0010) involving rs7975232 and smoking. We also used logistic regression to analyze the synergistic effect between VDR-rs7975232 and smoking, compared with non-smokers with rs7975232 -CA or AA genotype, smokers with rs7975232 -CC genotype had the highest risk of RCC. However, we did not find any significant synergistic effect combination in GMDR model of SNP and alcohol drinking interaction. Previously, the gene- environment interaction on RCC risk has been reported between VEGF gene and smoking in two studies [30, 31]. But to date, no studies reported the impact of interaction between VDR gene and smoking on RCC risk in Chinese populations. The significant interaction between rs7975232 and smoking indicated that the RCC susceptibility could be influenced by comprehensive factors, which including VDR- rs7975232, environment factors, such as smoking, and the interaction between rs7975232 and smoking. The biological mechanism of the interaction between rs7975232 and smoking is not very clear. We believe that the degree of association between rs7975232 and RCC risk is influenced by smoking status.

Although our study is important for us to understand the genetic susceptibility of RCC, there are still several limitations in this study. Firstly, this study only selected a few common SNPs, more SNPs should be included in the study. Secondly, more environmental factors should be included in GMDR model to explore more gene environment interaction combinations. Thirdly, the results of this study still need to be verified in other studies with larger sample size. Lastly, participants included in this study were all Chinese Han population, so the results obtained in our study need to be verified in different populations, ethnicities and SNPs in the VDR gene.

In conclusion, we found that both the A allele of **rs7975232** within VDR gene, rs7975232 and smoking were statistically associated with increased RCC risk.

## Data Availability

The datasets generated and/or analyzed during the current study are available from the corresponding author on reasonable request.
